# Recent Advances in Loop Heat Pipes with Flat Evaporator

**DOI:** 10.3390/e23111374

**Published:** 2021-10-20

**Authors:** Pawel Szymanski, Richard Law, Ryan J. MᶜGlen, David A. Reay

**Affiliations:** 1 Faculty of Mechanical Engineering and Ship Technology, Gdansk University of Technology, 80-233 Gdańsk, Poland; 2 School of Engineering, Newcastle University, Newcastle Upon Tyne NE1 7RU, UK; Richard.law2@newcastle.ac.uk (R.L.); David.reay@newcastle.ac.uk (D.A.R.); 3 Aavid, Thermal Division of Boyd Corporation, Ashington NE63 8QW, UK; Ryan.McGlen@boydcorp.com

**Keywords:** loop heat pipe, flat evaporators, porous structures, capillary pressure, nanofluids

## Abstract

The focus of this review is to present the current advances in Loop Heat Pipes (LHP) with flat evaporators, which address the current challenges to the wide implementation of the technology. A recent advance in LHP is the design of flat-shaped evaporators, which is better suited to the geometry of discretely mounted electronics components (microprocessors) and therefore negate the need for an additional transfer surface (saddle) between component and evaporator. However, various challenges exist in the implementation of flat-evaporator, including (1) deformation of the evaporator due to high internal pressure and uneven stress distribution in the non-circular casing; (2) heat leak from evaporator heating zone and sidewall into the compensation chamber; (3) poor performance at start-up; (4) reverse flow through the wick; or (5) difficulties in sealing, and hence frequent leakage. This paper presents and reviews state-of-the-art LHP technologies; this includes an (a) review of novel manufacturing methods; (b) LHP evaporator designs; (c) working fluids; and (d) construction materials. The work presents solutions that are used to develop or improve the LHP construction, overall thermal performance, heat transfer distance, start-up time (especially at low heat loads), manufacturing cost, weight, possibilities of miniaturization and how they affect the solution on the above-presented problems and challenges in flat shape LHP development to take advantage in the passive cooling systems for electronic devices in multiple applications.

## 1. Introduction

Loop Heat Pipes (LHPs) are high performance passive two-phase heat transport devices that allow the transport of heat over long distances or against high gravitational acceleration loads by the evaporation and condensation of a working fluid that flows around the loop. LHPs are electrical power free, high-reliability devices with flexibility and robustness in design and assembly as well as antigravity capability of heat transport over distances of up to 20 m. As such, the LHP offers many advantages compared with traditional cooling systems. LHPs utilize latent heat of vaporization of working fluid inside a loop to transport heat from a source to a sink, and to achieve this they take advantage of surface tension generated in a porous structure (a.k.a. “wick”) to create the capillary forces needed for the circulation of the fluid [1,2].

Understanding the mechanisms occurring in LHP and their components requires multidisciplinary knowledge of a number of issues, including two-phase heat transfer phenomena occurring in the entire loop, innovative manufacturing processes (in particular wick construction), metallurgy, chemistry, material science, capillary fluid flows, fluid dynamics, mathematical modelling, computer-aided design, imaging techniques and nanotechnology. Hence, the choice of the optimum and final design of LHP depends on many factors. Things to consider include overall thermal performance, heat transfer distance, robustness, reliability of operation at adverse tilts in gravity fields, acoustic issues, manufacturing cost, weight, integration into the end application and potential miniaturization requirements.

**Figure 1:**
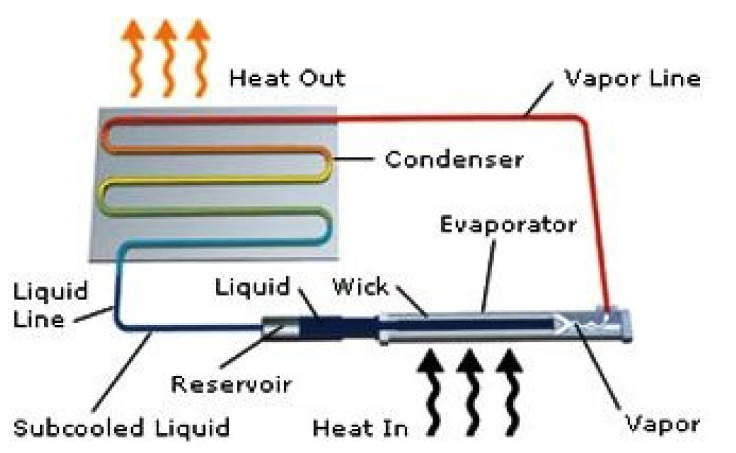
LHP Schematic Diagram Showing Main Components and Functionality [3].

Traditional LHP consists of five main components: evaporator, vapor line, condenser, liquid line, compensation chamber (CC) (i.e., “reservoir”). Typically, only the evaporator and CC contain a complex porous wick structure, while the rest of the loop is made of smooth wall transport lines. A schematic of the traditional LHP is presented in Figure 1.

The principle operation of the LHP is relatively simple: when the load is applied to the evaporator, the liquid is vaporized at the outer surface of the wick, and the menisci formed in the evaporator wick develop a capillary pressure to push the vapor collected in the vapor micro-grooves through the vapor line towards the condenser, where it condenses as heat is removed by the radiator. Then, the condensed liquid travels back through the liquid transport line to the CC (located at the evaporator core) and provides liquid replenishment to the evaporator wick to complete the circulation. As the capillary forces developed in the evaporator wick are the driving source for the circulation of the working fluid along the loop, no external power is needed in the operation of an LHP.

The LHP was developed in 1974 in Russia by Y.F. Gerasimov and Y.F. Maydanik [1]. This date is considered to be the beginning of LHP research and since then laboratories worldwide have been conducting advanced research on understanding LHPs’ operating characteristics. Notwithstanding, LHPs utilizing a flat-shaped evaporator is a fairly new idea, thus multiple laboratories worldwide are focused on investigating and exploring possibilities to construct and test modern LHPs with flat evaporators, with the aim of improving their performance. Two main directions have been noted in the development of flat evaporators, which may be arbitrarily separated into evaporators with opposite replenishment and longitudinal replenishment [4]. The flat-shaped evaporator can be considered the most advantageous design for compact enclosures as it provides possibilities of design miniaturization and integration into concealed, narrow areas. Flat evaporators have a significantly higher thermal contact area compared to traditional cylindrical evaporators, removing the need to attach the evaporator to an additional ‘saddle’ component, which is necessary for the traditional cylindrical-type evaporators, to allow for interfacing with the usually flat heat input surface. As a result, this provides an evaporator height and mass reduction, as well as a reduction in the overall thermal resistance of the LHP, as the conduction path through the conventional saddle is replaced with two-phase heat transfer [4,5,6]. LHPs, in general, offer many advantages in applications in aerospace (due to high-g capabilities and lightweight), space (due to lightweight, large condenser line length/radiator area), terrestrial ruggedized applications (due to slightly lower thermal specification, but out of the range of conventional heat pipes due to lift height, flexibility required to overcome vibration loading). The flat evaporator LHP has additional advantages including ease of component installation on the evaporator surface and uniform temperature distribution on the thermal contact surface caused by the even thermal path between evaporation interface and heater surface, which is especially important when cooling electronics. Further, in cases when the heat sources are closely packed, in some designs, there is the possibility to attach two symmetric thermal contact surfaces/interfaces (on two sides of the evaporator) in two heat-absorbing surfaces [4]. Due to the above advantages, especially for electronic cooling, the flat type LHP has been extensively investigated both theoretically and experimentally [4,5,6,7].

Despite all the above-presented advantages, various technical problems and challenges currently exist in the development of flat evaporators LHP, including:

− Sensitivity to internal fluid saturation pressure that can potentially cause stress, deformation and consequently the ballooning of the evaporator wall and wick, which can lead to the deterioration of the heat input surface contact with the heat source and loss of thermal connection between the heat input wall and the wick. The high positive saturation pressure created by certain working fluids in the evaporator may distort the evaporator casing shape or wick structure. Such a circumstance requires a more conscientious design of evaporator casing which might result in an increase in the wall thickness, increase mass or limit the choice of the working fluid, which may restrict the choice of casing materials [4,6].
− Increased heat leakage (i.e., “parasitic heating”) from the evaporator heating zone and sidewall into the compensation chamber (CC), which results in the increase of the CC temperature and consequently the LHP resistance and frequent failures in the start-up, especially at low heat loads. The construction of a flat-shaped evaporator requires installation of the heating zone very close to CC, which promotes parasitic heating from the evaporator to CC, therefore is a challenge to overcome. Furthermore, the flat-shaped evaporators have a larger sidewall area which facilitates conduction, resulting in a rise in CC temperature. This reduces the overall thermal performance of the LHP and may also cause a failure in the LHP start-up at a low heat load. A novel mechanical and thermal design of the evaporator can be considered to overcome this challenge [6]. For example, this effect can be reduced by: (1) increasing heat exchange intensity in the evaporation zone; (2) decreasing thermal resistance of the evaporator wall through which the heat load is supplied; and/or (3) by enhancing heat exchange to the working fluid at the wall-wick boundary.
− Increased heat loss through the wick into the liquid bore, causing a temperature rise of the liquid being supplied to the evaporator and consequently a higher operating temperature and change of start-up failure. Typically, the wick thickness is large, to minimize conduction through the wick [8,9].
− The difficulty of sealing the casing/wick structure due to relatively long, often square edges. This can cause leakage and consequently failure of the flat evaporator LHP operation [10,11,12];
− Difficult start-up at: (1) low operating temperature (due to low vapor pressure) [1]; (2) high g-loads or restarting after the high-g load period. High g-load conditions might cause a reverse flow of working fluid that influence LHP start-up and restart after start-up or situations where the working fluid stalls in the condenser, causing the onset of evaporator dry-out; (3) when LHP is orientated against gravity, that affects the liquid charge and CC size.

To solve the above-presented challenges, multiple laboratories around the world endeavor to find novel manufacturing methods, designs and construction materials to develop or improve the LHPs construction to take advantage of the passive cooling systems for electronics in multiple space and terrestrial applications. It includes the following main principles:

− Customizing of new wick properties and construction of new wick profiles to build ultra-performance LHP designs, understanding the manufacturing process;
− Maximizing the distance of the liquid motion in the wick;
− Organization of effective heat exchange during the evaporation and condensation of the working fluid;
− Maximizing the heat transport distance.

Hence, this review is focused on presenting and reviewing state-of-the-art technologies and how they affect the solution on the above challenges in flat shape LHPs development.

## 2. Novel Wick Materials, Wick Properties and Wick Construction

While affecting the performance of LHP fundamentally, the wick is the most important yet most difficult component to fabricate in the LHP. Since the beginning of the LHP development, many researchers have worked to improve the performance of porous wick structures, which is determined by pore sizes and porosity: finer pore size wicks can provide the LHP with higher capillary forces; with higher porosity, the permeability of wick is larger, which means less resistance improved the for vapor and liquid distribution into the wick.

As outlined in the sections below, various researchers have focused on wick enhancement in the following areas: Bi-porous wicks, additively manufactured (AM) wicks or improvement in micro/nanostructure of the wick.

### 2.1. Bi-Porous Wicks

**Figure 2:**
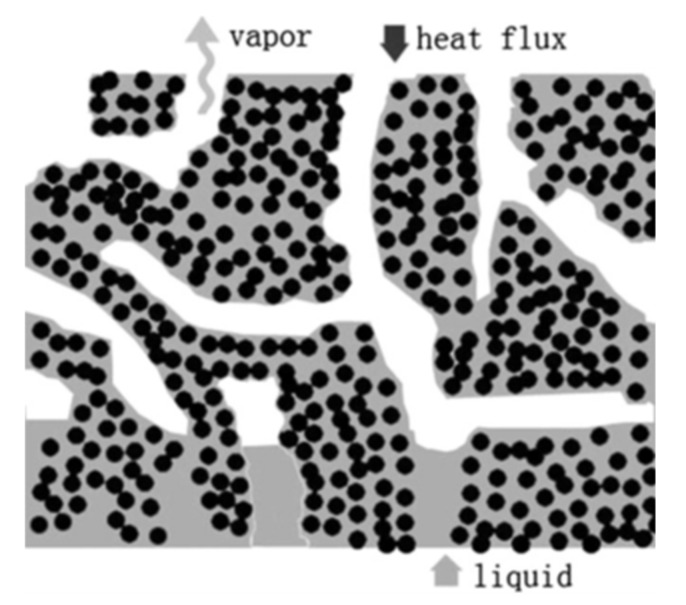
Schematic of the bi-porous wick [14].

In 2009, Yeh et al. [13] found that porous (traditional single porous) wicks, at high heat fluxes, were intolerant of boiling and easily occupied by the vapor to form a vapor blanket inside the wick, raising the thermal resistance between the wick and evaporator wall and reducing the ability to remove high heat fluxes from smaller evaporator surfaces. To respond to this challenge bi-porous wicks were proposed, whereby the wick contains two different pore sizes within its structure, providing higher permeability and lower thermal resistance wicks in comparison to single-porous wicks. As shown in Figure 2, the two different size pores can allow the wick to achieve better performance through the separation of liquid and vapor: the large pore can reduce liquid flow resistance effectively, moreover, large pore also provide extra area for the liquid film to evaporate, and the small pore can maintain the system with sufficient capillary force. When the LHP operates in the high heat flux condition, the vapor will selectively occupy the large pore, forming a vapor channel. The meniscus in the wicks includes two parts: one within in the large pore, and one within in the small pore. The vapor channels in the large pores provide extra area for film evaporation. Moreover, the existence of the large pores makes it easier for vapor to secede from the wick. Two different pore sizes inside the single wick structure ensure better performance due to separation liquid and vapor phases: that is, small pores inside the wick provide appropriate capillary forces, and large pores can reduce liquid flow resistance and provide a sufficient evaporating area for liquid.

The biggest advantages of such wicks are that the large pores provide additional area for the liquid film to evaporate and makes it easier for vapor to leave from the wick and flow into the vapor transport line, reducing the vapor blanket layer and its impedance on the returning liquid flow. The small pores provide LHP with enough capillary force and continue to function as liquid supply routes and increase the evaporative surface area [15]. While early heat transfer experiments of bi-porous wicks can be traced back to 1984 [16], Yeh et al. presented the first successful LHP (traditional shape) with bi-porous wick and proved that bi-porous wick improves the heat transfer capability of LHPs and that the heat transfer coefficient of the bi-porous wick is several times higher than the relevant single-porous wick at the same heat transfer conditions (precisely: the evaporative heat transfer coefficient of the better bi-porous wick reaches a maximum value of 64,000 W/m^2^ K, which is approximately six times higher than that of the monoporous wick).

Bi-porous wicks provide higher porosity. It not only decreases the effect of heat leak through the bi-porous wick to the compensation chamber in the design of an LHP evaporator with the ‘‘inverted meniscus scheme”, but can also increase the surface area for liquid film evaporation. Because the large pores were also generated by dissolving the pore formers in bi-porous wicks, the pore sizes are easier to control than those passively formed by collecting the clusters of small porous particles in bi-porous wicks.

**Figure 3:**
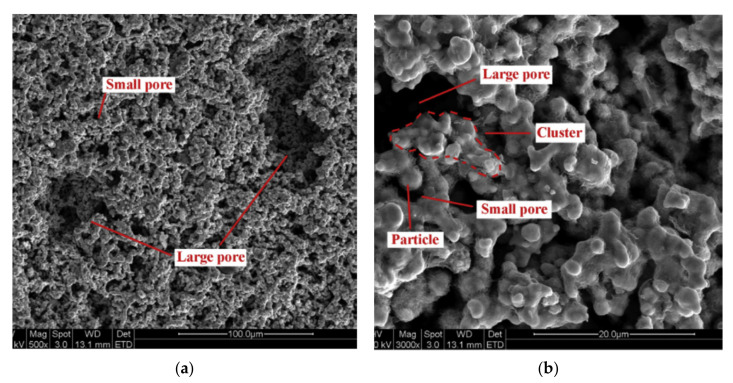
Magnified images of bi-porous wicks (**a**) SEM photograph ×500 (**b**) SEM photograph ×3000 [17].

Chen et al. in 2012 presented the first successful design of a flat-shaped LHP evaporator with a bi-porous wick and proved it is possible to start-up at low power (2.5 W). The maximum heat load was 130 W at the allowable evaporator temperature of 60 °C. The LHP showed a very fast response to variable heat load and operated stably without obvious temperature oscillation [17]. In 2012 Li et al. presented two different methods (cold pressing sintering and loose powder sintering) to manufacture bi-porous wicks. It was proven that the flat LHP can start-up and run reliably under different heat loads and can operate stably and reliably in high heat flux conditions [18]. Liu et al., (2012) proved that the start-up time of flat shape LHP with a bi-porous wick is short. The LHP can start-up at low heat loads (20 W) and the start-up time of LHP decrease with the increase of heat load applied to the evaporator [14]. Wu et al., (2015) investigated the effect of the powder-mixing parameter in bi-porous wick manufacturing on the intensification of loop heat pipe performance. In this study, polymethyl methacrylate (PMMA polymer) was first used for wick manufacturing as a sacrificial layer for large pore formations. Performance testing indicated that, compared with using a mono-porous wick, heat transfer performance was enhanced by 50% results also indicated that larger powder size leads to better vapor transport and evaporation, but beyond a certain point the large pores can cause a weakened structure [19]. Kumar et al., (2018) presented the application of bi-porous wick in traditional circular LHP thermal-fluidic transport characteristics of bi-porous wicks for potential loop heat pipe application. According to the authors, the evaporative heat transfer coefficient at the wick interface decreases with an increase in input heat loads. This is attributed to the increase in thermal resistance with applied heat loads as the vapor zone under the heated fin enlarges with an increase in heat loads [20]. Zhang et al., (2020) presented LHP with a bi-porous wick and a flat disk evaporator that can transport heat for a distance to up to 1.6 m. The author presented that the LHP can start up successfully also at very low power (2.5 W) [21]. Table 1 presents a comparison between recent works related to bi-porous wick LHPs. Figure 3 presents the example photographs of a magnified image of bi-porous wicks [17].

To summarize, a bi-porous wick applied in flat evaporator LHPs improves the performance of the porous wick structure, improve heat transfer capability of LHP, decreases the effect of heat leak through the bi-porous wick to the CC and improves the start-up time at a low operating temperature or low operating power. The drawback of the bi-porous wick is their cause to create a reverse flow of vapor across the wick and hence it is necessary that they have a sufficient thickness.

### 2.2. Additive Manufactured Wicks and LHPs

**Figure 4:**
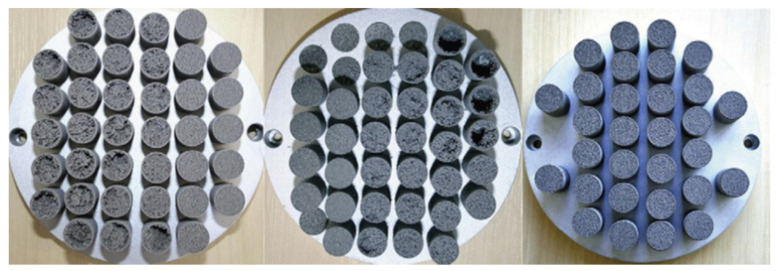
Porous samples made for permeability measurements [25].

**Figure 5:**
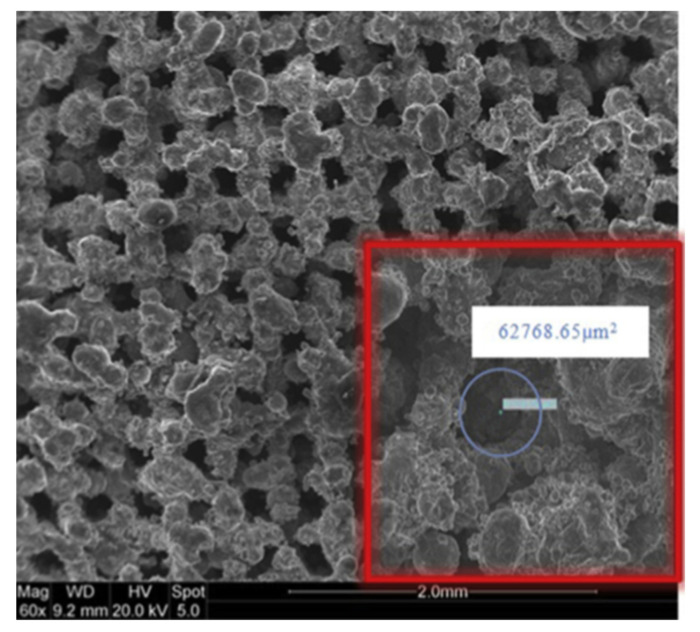
Magnified image of regular SLM porous structure [12].

**Figure 6:**
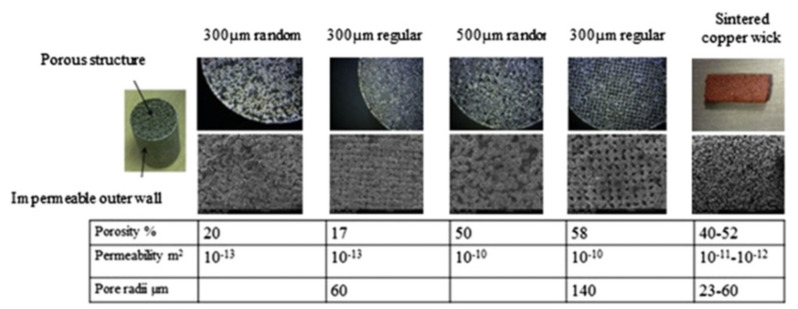
Comparison of the SLM porous structure measured properties with those of a conventional sintered copper wick [12].

A novel approach to overcoming deformation (ballooning) in the flat evaporator is to utilize advanced, additive manufacturing (AM) techniques colloquially known as 3D printing (3DP) to incorporate structural elements within the evaporator and wick assembly that reinforce the device, thereby preventing ballooning. AM heat pipe technology was pioneered by McGlen and Sutcliffe in 2018, who utilized AM technique to construct titanium-ammonia heat pipes with integrated micro-scale lattice capillary wick structures [22,23]. This technology allows the development of a device with complex geometry and high surface area to volume ratio (A/V) in order to maximize the interaction between the heat source and heat sink or to maximize the surface area for evaporation/condensation processes [24]. The most popular technology for developing heat transfer devices is selective laser melting (SLM). This technology allows the fabrication of products with a lower cost-to-complexity ratio and quicker production time compared to other manufacturing processes and gives the possibility of producing customized and complex freeform shapes which are in LHPs [10]. Ameli et al., (2013) developed the first aluminum/ammonia heat pipe with a sintered wick structure where samples have been manufactured using the SLM technique with various wick characteristics. The authors proved the capability of producing very complicated wick structures with different thickness, porosity, permeability and pore sizes in different regions of a heat pipe in addition to the solid (nonporous) walls while the entire heat pipe can be produced in a single process. The view of the samples made for permeability measurements presented in Figure 4, magnified image of the sample presented in Figure 5 and comparison of the SLM porous structure measured properties with those of a conventional sintered copper wick presented in Figure 6 [25].

**Figure 7:**
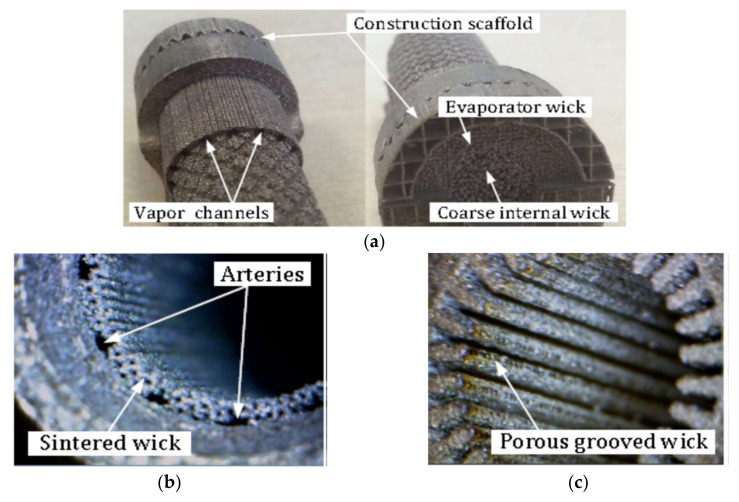
AM wick sample for (**a**) LHP together with close up on varied density wick structure; (**b**) AM Aluminum–Ammonia HP with a sintered hybrid wick structure, arterial wick (**c**) porous grooved wick (HP: ∅14 mm and 70 mm length) [23,28].

It should be noted again that one of the biggest challenges in flat LHP construction is the above-mentioned sealing casing/wick structure due to the relatively long edges and that improper sealing causes leakage and consequently the failure of the LHP. Using SLM will reduce this problem as the LHP elements are made in a layer-by-layer sintering process that selectively melts powdered metal thus AM parts can provide a hermetic seal and consequently prevent the back-flow of vapor directly to the compensation chamber. The development of AM LHPs enables complex mechanical designs and offers an increased level of integration of the two-phase thermal management system (TMS) into the chassis elements and enables direct thermal management of the electronics components. An additive manufactured LHP evaporator was demonstrated by McGlen and Sutcliffe [22,23]. The device demonstrates additive manufacture of a single evaporator component incorporating a primary wick with embedded vapor flow network, a larger pore size secondary wick representation, solid internal vapor flow bulkheads and a solid external sleeve. The views of AM wick are presented in Figure 7.

The other benefit of using SLM technology for LHP production is the possibility of manufacturing a very efficient LHP wick. The SLM technology controls the geometric size of the internal structure of the wick aiming to achieve an optimal design according to the specified requirements. Estarte et al., (2017) constructed a traditional cylindrical-shaped LHP with a primary wick fabricated in SLM technology. This wick has an 80 µm pore radius and a whole LHP was able to transfer 80 W [26,27]. Anderson et al., (2017–2021) constructed a cylindrical LHP using AM method where the envelope, primary wick, and secondary wick were 3D printed in a single process. This assembly reduces the risk of leakage of LHP and eliminates a knife-edge-seal.

**Figure 8:**
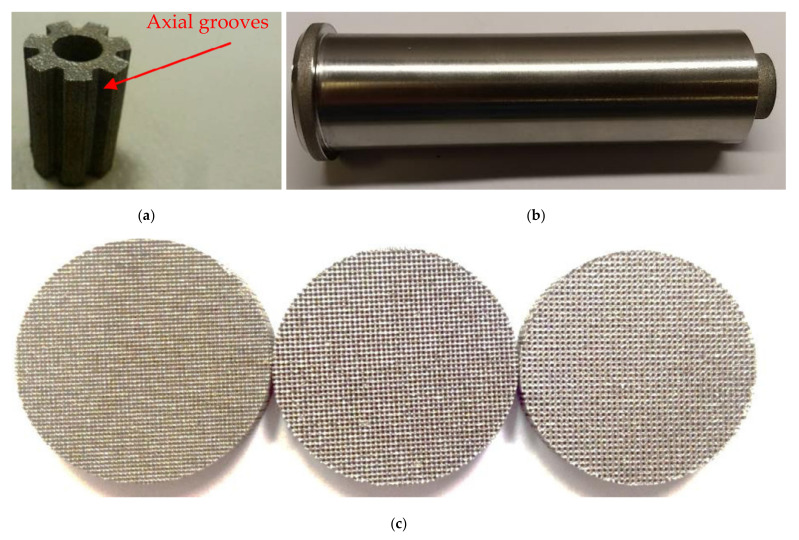
Porous structures fabricated through additive manufacturing for the needs of LHP: (**a**) Esarte et al. [26] (**b**) Richard et al. [11] (**c**) Hu et al. [29].

The author constructed an LHP with AM wicks of 4.9 µm to 62.8 µm pore radius. The author presented AM LHP successfully and robustly, operating in adverse elevation in multiple angles that can transfer up to 350 W and the maximum heat transport distance reached in one of the tests was about 3.2 m, however, it was not indicted which pore size this particular LHP test piece was constructed from. Moreover, the author proved that 3D printed evaporators can significantly reduce the overall cost of the entire device by eliminating costly labor-intensive processes associated with multiple machining steps. The LHP was made by 316LSS and ammonia was used as the working fluid [11,12,27,28]. Hu et al., (2020) constructed the first flat LHP with the AM wick in an application in the chemical reactor. The authors made stainless steel wicks with pore diameters of 108 µm, 208 µm and 324 µm and used deionized water as a working fluid. The authors indicated that this LHP could start successfully in about 100 s at a low heat load of 20 W (2.83 W/cm^2^) and could stably operate in a wide range of heat loads from 20–160 W (22.63 W/cm^2^) [29]. The porous structures fabricated through additive manufacturing for the needs of LHP are presented in Figure 8. The table presents a comparison between recent works using AM technology in manufacturing LHPs or LHP wicks presented in Table 2.

AM is a very promising method of wick or LHP manufacturing but a lot of research still needs to be carried out in this area. The main drawback is currently the minimum pore size that can be manufactured using AM, which limits the use of AM LHPs in high g/long transport distance applications. However, this technology is currently under development and it is hoped that in the near-to-medium term AM can be utilized to overcome multiple challenges in LHP production, such as quicker production time, manufacturing more sophisticated, advanced and customized LHPs, and what is most important for resolving a frequent problem with sealing casing/wick structures in flat evaporator LHP.

### 2.3. Wick Surface Treatment

Another conception to enhance LHP heat transfer performance, thermal conductivity, wettability and hardness of the wick is to improve the porous wick efficiency by altering the micro-nanostructures of the wick. Thus, in the last twenty years, various attempts have been made to develop such a structure (e.g., microporous coating [30], nanoparticle deposition [31], carbon nanotube coating [32], nanowire array [33], micro/nano ridges [34], micropillars [35], micro-cavities [35], nano-cavities [35], nanofibers [36] metallic nanowire coating [37,38,39] or adding a gold layer on the wick external surface [38]). Due to the enhanced capillary pumping effect and improved liquid spreading, the maximum heat fluxes dissipated by LHPs increased significantly. Recent work reports that the critical heat fluxes dissipated by LHP have been increased to 100–200 W/cm^2^ while the heat transfer coefficient can approach 200 kW/m^2^K [37]. Furthermore, Guo et al., (2020) showed that adding a micro-nanostructure layer to the particle surface of the wick increased surface roughness and surface area which resulted in the improvement of the capillary action of the wick. Adding the micro-nanostructure layer to the particle surface of the wick also improved LHP start-up at lower heat loads and LHP has smaller temperature fluctuations in comparison to traditional LHP. The nanostructures on the wick’s surface also provide more nucleation sites and a large surface area for phase change heat transfer. The porous wick with the micro-nanostructure layer has a strong liquid suction capacity and ensures a sufficient liquid supply to the heating surface at a high heat flux, thus delaying the occurrence of the dry-out phenomenon [40].

The other new invention enhancing the thermal conductivity, wettability and hardness of the wick are presented by Giraudon et al. [38]. The authors coated a wick sample by adding a very thin gold layer (thinner than 0.1 μm) on the wick external surface and analyzed the influence of this layer on surface properties, maximum heat load and heat transfer coefficient. The results show that this procedure enables enhancement of the evaporator thermal performance and decreases the thermal contact resistance between the evaporator wall and the wick since the gold has a very high thermal conductivity and low hardness. Further, the gold improved the wettability of the wick, delaying the occurrence of boiling inside an evaporator.

To summarize, the treatment of the wick micro/nanostructures can enhance the wettability of the wick and hence improve a liquid spreading inside a wick and capillary pumping effect, which leads to the improvement of LHP thermal conductivity, maximum heat fluxes dissipated by LHP and maximizes heat transfer distance. Such a treatment improves the hardness of the wick and hence prevents the deformation of flat evaporator LHPs. This addresses multiple challenges in the production of novel flat evaporator LHPs.

### 2.4. Non-Metallic and Composite Wicks

**Figure 9:**
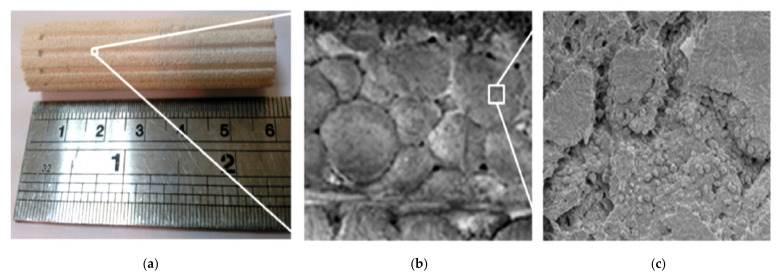
PTFE wick (**a**) general view and SEM images (**b**) 300 µm zoom (**c**) 10 µm zoom [46].

**Figure 10:**
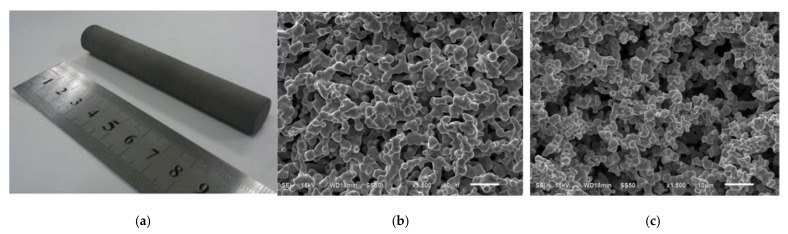
Composite wick (**a**) general view and SEM images of the pore structure of (**b**) pure Ni layer (**c**) Ni–10 wt% Cu layer [48].

**Figure 11:**
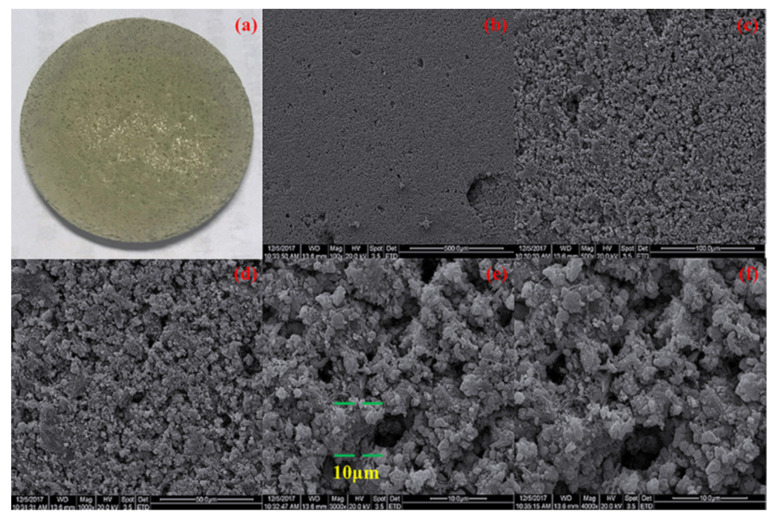
Surface morphologies of the pouring surface at different magnifications, (**a**) the pouring porous wick image; (**b**) ×100; (**c**) ×500; (**d**) ×1000; (**e**) ×3000; (**f**) ×4000 [50].

**Figure 12:**
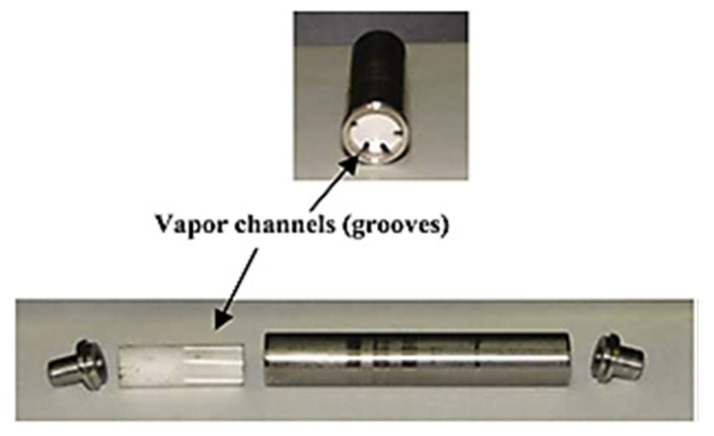
Ceramic wick with vapor channels (grooves) used in the LHP [45].

To reduce the heat leak from the evaporator heating zone and sidewall into the CC, several attempts have been made in the flat evaporator LHP research area. As mentioned above, a large heat leak can increase the LHP operating temperature, make it difficult to start up the LHP and is one of the biggest challenges in flat evaporator LHP production. One of the ideas for solving the parasitic heating problem in flat evaporator LHPs is to select a non-metallic wick material such as silicon, ceramic, composite and polytetrafluoroethylene (PTFE) [41,42,43,44,45,46,47,48,49,50,51]. Owning their low thermal conductivity, these non-metallic materials wicks could significantly reduce a heat leak in flat LHP. However, this low thermal conductivity can also be of detriment to heat transfer in the wick, and careful design is necessary. Thus, nowadays a lot of LHPs use metal as a wick material. However, Wu et al. [46,51] found that the LHP system with a PTFE wick compared to the nickel wick while having comparable performance resulted in a reduced parasitic heat leakage and a lower operating temperature. Moreover, LHP with a PTFE wick could reach a critical heat load of 600 W, while that of the LHP with a nickel wick was only 500 W. Therefore, the applications of the non-metallic wicks in high heat transfer capacity cooling devices still need more studies to be validated. Xin et al. [48] presented an LHP with a composite wick having two different effective thermal conductivities as a solution of the heat leak problem, that is, wick has a higher thermal conductivity on the side close to the vapor channels and lower thermal conductivity on the side close to the liquid in the compensation chamber. The wick was constructed with the higher thermal conductivity on the side close to the phase change region and the lower thermal conductivity on the side close to the liquid in the compensation chamber, which helped to increase the power absorbed by the working fluid for phase change and prevent heat leak from the evaporator to the CC. However, the LHP presented by Xin et al. is circular (traditional), but such a concept could also be applicable in flat evaporator LHP to meet the above-presented challenges, prevent parasitic heating and hence increase a startup time and lower operating temperature. The comparison between different attempts of heat leak prevention by using non-metallic or composite wicks is presented in Table 3. The figures below present views and SEM images of the PTFE wick (Figure 9), composite wick (Figure 10), pouring wick (Figure 11) and ceramic wick (Figure 12).

## 3. Working Fluid

In addition to the design of the LHP elements, to maximize LHP performance the proper selection of working fluid is extremely important. The choice of working fluids for most modern flat LHPs is realized on the same criteria as for conventional LHP. Traditionally, working fluids have been categorized as either cryogenic fluids such as helium, neon, oxygen and nitrogen; moderate-temperature fluids such as methanol, ethanol, ammonia, acetone and water; or high-temperature liquid metal fluids such as potassium, lithium or sodium [1]. The choice of working fluid must fulfil the following criteria: (1) compatibility with wick and wall materials; (2) good thermal stability; (3) high wettability of wick and wall materials; (4) correct level of vapor pressures over the LHP operating temperature range; (5) high latent heat; (6) high thermal conductivity; (7) low liquid and vapor viscosities; (8) high surface tension (9); correct freezing point. Moreover, in LHP application, it is desired that working fluid has a high value of surface tension (σ) in order to generate a high capillary driving force (ΔP = 2σ/R) and enable the LHP to operate against gravity [52].

### Nanofluids

One of the most advanced methods to improve the thermal conductivity of the LHP working fluid is using Nanofluid (nanoscale solid particles mixed with plain fluid) as the thermal conductivity of solid materials is higher than fluids, therefore the mixture will have a higher overall thermal conductivity. Gunnasegaran et al., (2013) presented the first flat LHP using nanofluid as a working fluid. This LHP in which the setup consists of a separated tank with a pump was filled with silica nanofluid (SiO_2_–H_2_O). The author performed thermal tests of flat LHP using pure water and nanofluid at various heat loads. The results showed the positive influence of nanofluid utilizing as an LHP working fluid on the system thermal performance. LHP with silica nanofluid also yields a lower temperature and reaches a steady state faster than LHP using pure water [53]. The table presents a comparison between recent works of using nanofluids in LHPs presented in Table 4.

To date, many researchers proved a successful LHP operation with different working fluids such as Cu–water [55], Al_2_O_3_–water [54], graphene-water [56] and proved that nanofluid increase the heat transfer performance of the LHP. The disadvantages of using a nanofluid in LHPs are (1) the need to use a wick with a relatively large pore diameter and hence the reduction of the pumping power of the wick due to a greater pressure drop as compared to pure liquid to gain equivalent heat transfer intensification, (2) long-term fluid settling and potential clogging of pores and flow passages, (3) possible damage of LHP elements by erosion, and (4) high cost of nanoparticle suspension. As of today, there is no life test data, or data about the application of LHP with nanofluid in space application and no data about the behavior of LHP with nanoparticles working in the high-g environment or future environmental costs of nano-particles being released into the environment, by accident or at end-of-life.

It should be noted that the above Section 2 and Section 3 analyzed current developments of wick material, wick properties, wick construction, novel manufacturing methods and novel working fluids and their influence on all LHPs (both flat and cylindrical evaporators), but the experience of the authors can be especially useful to solve above-presented problems and challenges in flat shape LHPs, what is the subject of this paper.

## 4. Modification in Construction of LHP

**Figure 13:**
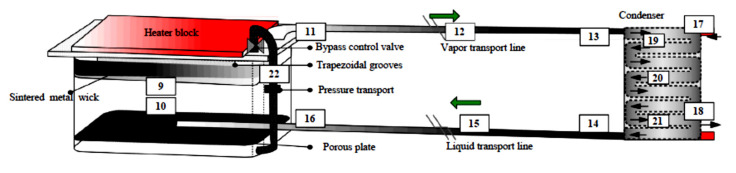
Schematic of flat LHP with a bypass line connecting vapor line and CC according to Boo and Jung [8].

**Figure 14:**
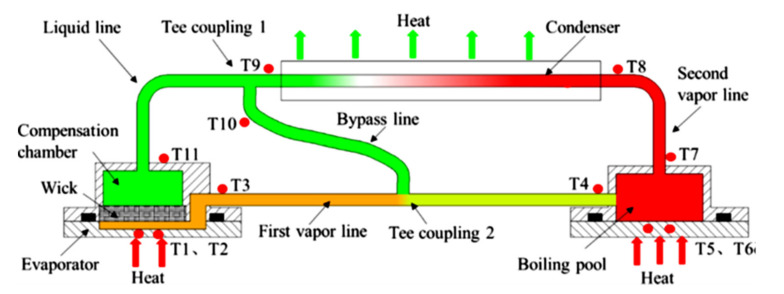
Schematic of flat LHP with bypass line connecting vapor line and condenser outlet according to Liu et al. [9].

Another challenge in constructing a flat LHP is to overcome start-up difficulties caused by the temperature overshot during the start-up period, especially at a low heat load. The physical mechanism associated with LHP start-up has been analyzed and experimentally investigated in detail according to the multiple factors and operating conditions affecting these phenomena [57,58,59,60]. Successful start-up should happen immediately after a heat flux is applied to the heating area of the evaporator. However, a start-up can have a time delay, which can lead to the overheating or damage of the cooled electrical device. A successful start-up depends on how fast the required temperature difference can be produced between the liquid in the CC and the liquid line outlet and the vapor generated in the evaporator and vapor line inlet. During the start-up period, the temperature at the interface between the heater and evaporator wall may rise to a level much higher than that in the steady-state condition, and it could exceed the maximum allowable value. Such a phenomenon is called a temperature overshoot. Several technical solutions have been suggested to address this common drawback. Boo and Jung [8,60] proposed a bypass line between the vapor line inlet and the CC to boost the start-up of a flat LHP, especially for low thermal loads. A schematic of flat LHP with a bypass line according to Boo and Jung is presented in Figure 13. Such a solution can significantly reduce the minimum required thermal load necessary for the start-up. The experimental results for the LHP with the bypass line showed that the evaporator wall overshoot temperature was cut off as much as 92.5 °C and the start-up time was reduced by 5.2 min, without considerable sacrifice in thermal resistance (in comparison with the same LHPs without the bypass line). It was also observed that the bypass line can aid a quick escape of the vapor from the evaporator when the thermal load is sufficiently high, such that the main loop flow could discharge the imposed thermal load via the condenser. The only drawback of this solution is that a mechanical bypass control valve and control electronics are required in this system, which might reduce LHP reliability, especially in space applications. Liu et al., (2020) indicated that the bypass line, installed between the condenser outlet and the vapor line, accelerates the return of working fluid, reduces the adverse effect of the heat leakage and prevents the dry-out of the evaporator boiling pool effectively. A Schematic of flat LHP with a bypass line according to Liu et al. is presented in Figure 14. Such an LHP construction modification can eliminate a temperature overshoot. Furthermore, the installation of the bypass in LHP can slightly reduce the evaporator heating wall temperature and reduce an LHP thermal resistance by more than 10% under various heat loads (in comparison with the same LHPs without the bypass line) [9].

**Figure 15:**
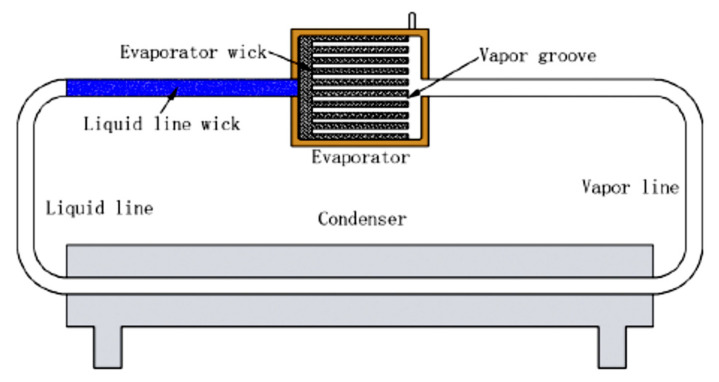
Schematic of flat LHP without CC [61].

Another interesting flat LHP construction modification is the design presented by Du et al., (2020), where the authors presented and tested a flat LHP without CC, as shown in Figure 15. The traditional CC design is substituted by accommodating the CC volume within the liquid return line. In this LHP design, the liquid line is fitted with a sintered wick to enhance start-up and steady operation capacity and reduce an evaporator size. The other benefit of covering the liquid line by the sintered wick is that this impedes vapor permeation and heat leakage in all operational conditions and the fluid in the inlet of the liquid line wick always stay at subcooled condition, which is beneficial for a successful start-up and steady operation of LHP. This LHP is sensitive to the change of heat load and has good self-regulation ability. The results show that the LHP can start up smoothly at any heat load and the highest temperature of LHP does not exceed 90 °C [61].

**Figure 16:**
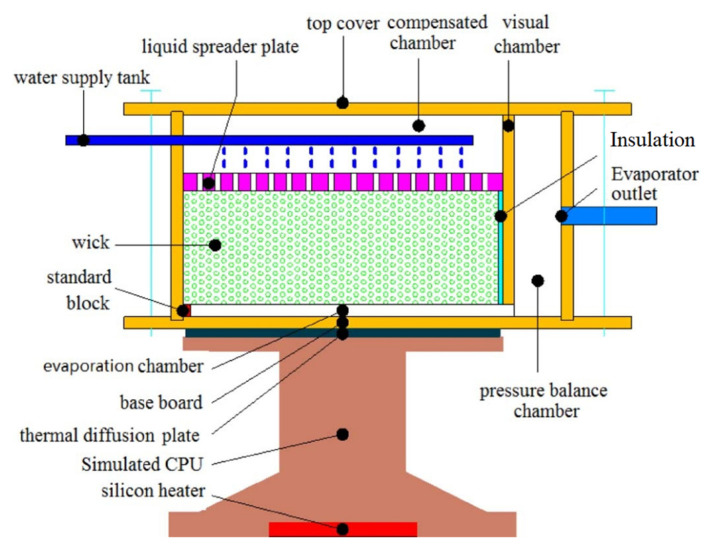
Schematic of the flat type evaporator with wick separated from the heating surface [62].

To eliminate a parasitic heat leak in the evaporator and to reduce circulation flow resistance Wang et al., (2016) [62,63,64] proposed a novel flat type evaporator with the wick separated from the heating surface, where the circulation inside LHP is mainly driven by the phase change formed at the vapor-liquid interface, unlike a traditional LHP where the pumping force is provided by the capillary wick. The schematic of the evaporator where the heating surface had no direct contact with the wick is presented in Figure 16. The biggest advantage of this design is the utilization of the new heat transfer mechanism to drive the vapor generated in the room between the wick and the evaporator wall for the circulation of the working fluid. The space between the wick and the bottom of the evaporator prevents the boiling phenomenon in the wick, thus the shear flow resistance generated by the phase change and the partial pressure of the vapor in the wick was almost eliminated. It has been designed as a buffer space—the pressure sharing chamber at the outlet of the vapor chamber, to control the working fluid in the vapor chamber realizing the unidirectional phase of liquid to vapor and to avoid the phase change phenomenon in the wick. The new flat type LHP was able to start fast and operate stably at a high heat load.

## 5. Miniature Flat LHP

With the recent development of compact electronic devices (e.g., notebooks, tablet computers, or smartphones), the flat type LHP can be considered the most advantageous design for compact enclosures with a big potential for design miniaturization and the possibility of dissipating high heat fluxes. However, to date, very few research laboratories make attempts at the successful miniaturization of flat LHPs working especially under natural air convection. The big challenge in the construction of a miniature LHP is generating the required temperature and pressure drop required for start-up and operation using a relatively thin wick. There are also strict and special requirements for thermal management of compact electronic devices, that is, (1) operation under natural convection without any active cooling implemented, (2) stable start-up at a low heat load, (3) case temperature below 85 °C at its full load in operation, (4) insensitive to gravity [65].

**Figure 17:**
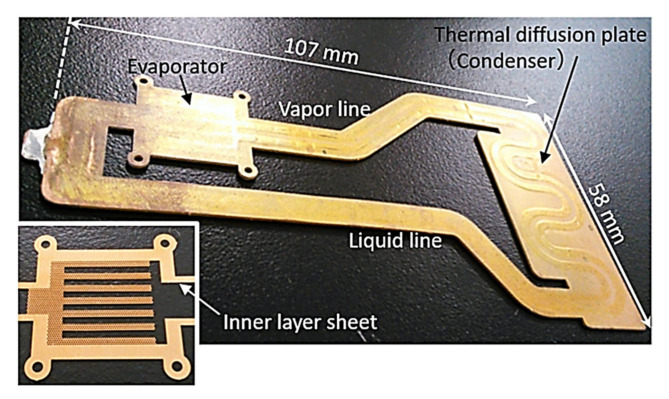
A prototype model of a miniature LHP [67].

**Figure 18:**
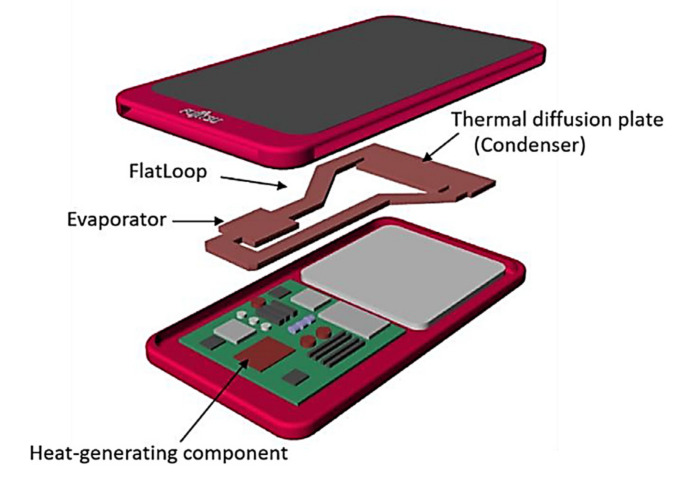
Concept of a smartphone equipped with miniature LHP [66].

Zhou et al., (2016) [65] presented a novel miniature copper-water LHP with a flat evaporator for cooling compact electronic devices, that can meet the above-presented requirements. This miniature LHP has a flat evaporator with a thickness of 1.19 mm that operates under natural convection, demonstrate a stable start-up at the heat input of 2 W with the evaporator temperature of 43.9 °C and works efficiently under different orientation (including antigravity). The minimum thermal resistance of 0.111°C/W was achieved at 11 W. This LHP can transport a maximum heat load of 12 W for a distance of about 105 mm. In 2020 Shioga et al. proposed a thermal management concept of installing an ultrathin LHP into a smartphone. The designed LHP had a thickness of 0.6 mm and 0.4 mm and was manufactured using a chemical-etching and diffusion-bonding process on thin copper sheets. This LHP facilitates heat dissipation by transporting the heat generated from the electronic elements to relatively low temperatures in small and thin electronic devices without using external electrical power. This miniature LHP worked efficiently under different orientations (as well as antigravity) and was a stable start-up at a heat load of 2 W. An LHP of 0.6 mm thickness achieved a thermal resistance between the evaporator and the condenser of 0.11 K/W for horizontal orientation, 0.03 K/W for a bottom heat orientation, 0.28 K/W for a top heat orientation was obtained at 20 W. An LHP of 0.4 mm thick achieved a thermal resistance of 0.21 K/W at an applied heat input of 7.5 W, which corresponded to a heat flux of 3.3 W/cm^2^. The prototype of this miniature LHP is presented in Figure 17 and the conceptual design is presented in Figure 18 [66,67].

**Figure 19:**
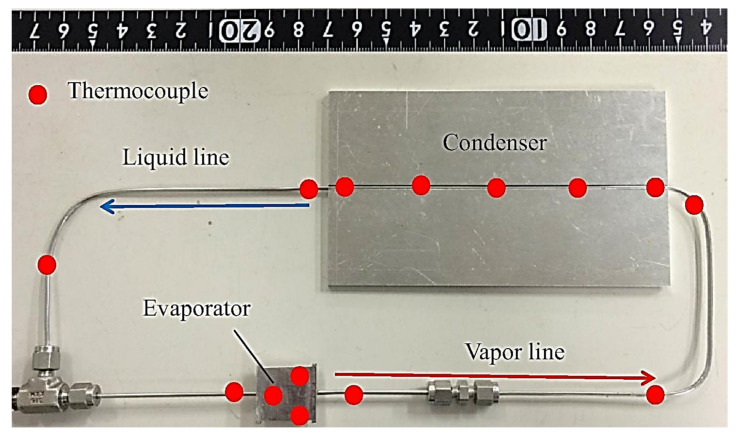
Photograph of miniature LHP [68].

Fukushima and Nagano in 2017 presented an LHP with an evaporator size of 20 mm × 10 mm × 3 mm (thickness) and a transport distance of 200 mm. The evaporator wick was made of a porous PTFE. The maximum heat load obtained by this LHP was 11 W and the minimum thermal resistance was 1.21 °C/W. This LHP could work under natural convection without any active cooling implemented; start-up stable at a heat load of 2 W. The LHP was made of aluminum and the working fluid was ethanol [68]. The photo of this miniature LHP is presented in Figure 19.

**Figure 20:**
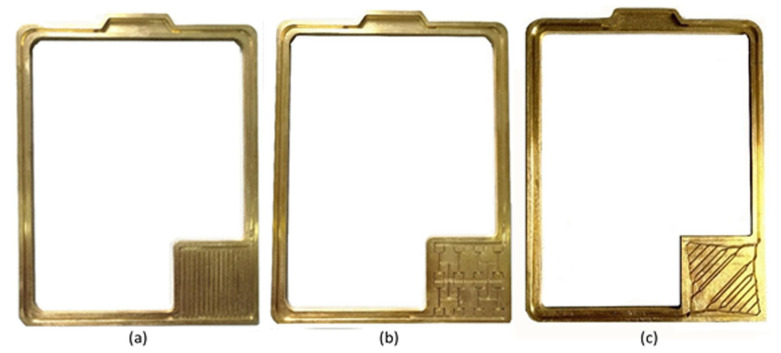
(**a**). The bottom part of the parallel microchannel loop heat pipe; (**b**). The bottom part of the self-similar fractal microchannel loop heat pipe; (**c**). The bottom part of the dendritic bionic microchannel loop heat pipe [69].

In 2020, Zhang et al. manufactured and experimentally investigated three wickless microchannel evaporator flat-type LHPs; that is, parallel microchannel evaporator, the self-similar fractal microchannel evaporator and dendritic bionic microchannel evaporator to present its potential and provide guidelines for further research on the design of microchannel evaporator of wickless miniature LHPs. The overall evaporator size was 52.5 mm × 52.5 mm and 2 mm thickness. The LHP was made of brass and used deionized water as a working fluid. The microchannel flat LHPs were tested according to start-up time, start-up temperature, operating temperature and thermal resistance. Such LHPs can achieve stable start-up at the low heat input and can successfully work in different gravitation orientations (precisely 5 inclined angles 0°, 30°, 45°, 60° and 90°). The maximum obtained power input was 10 W [69]. The photo of these microchannel LHPs is presented in Figure 20.

**Figure 21:**
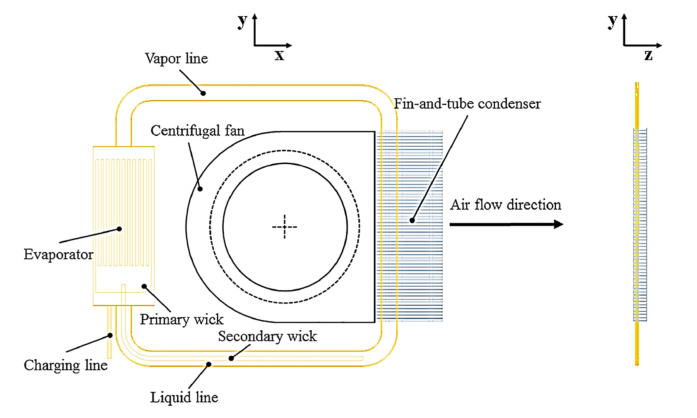
Schematic of miniature LHP [70].

**Figure 22:**
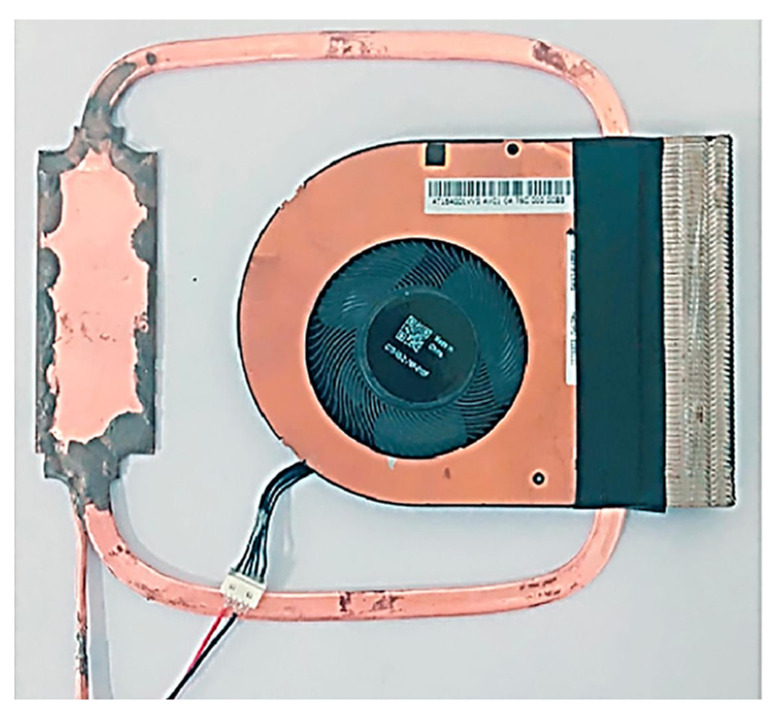
A photo of miniature LHP [70].

Zhou et al. in 2019 developed a 1 mm thick micro LHP module with a cooling capacity of 30 W and a heat transport distance of 132 mm in the application in ultra-slim laptop computers. The authors investigated whether this miniature LHP can operate under natural air convection and forced air cooling conditions with different fan voltages. Under natural convection the LHP can efficiently dissipate a heat load up to 10 W in all gravitational orientations, with a case temperature below 85 °C, however, the maximum heat load obtained under natural convection was 15 W with the casing temperature of 96.6 °C. Under forced convection, the miniature LHP can dissipate up to 30 W. The lowest system thermal resistance obtained was 2 °C/W at 25 W. The results indicate that by using the proposed module, cooling energy savings of up to 80% could be realized compared to the current applied miniature heat pipe module in a laptop computer [70]. The schematic of this miniature LHP is presented in Figure 21 and a photo is presented in Figure 22.

To summarize, the ultra-thin flat type LHPs presented above with thicknesses ranging from 0.4 to 3 mm, present stable start-up at a low temperature or low power and show potential to be applied for the thermal management of small, thin electronic devices, such as tablet and smartphones, where the heat transfer from heat-generating components is a major issue. The major challenge will be to take them from the lab scale to volume production.

## 6. Conclusions

LHPs with flat evaporators have been developed as an improvement to LHPs with cylindrical evaporators, as they provide a two-phase heat input interface with the entire heat source, as opposed to conventional circular LHP’s that have a conduction path through a metal saddle. This potentially reduces the weight, size and thermal resistance of the overall LHP cooling system. Furthermore, flat evaporator LHPs offer many advantages in the thermal management of multiple space and terrestrial applications. Recently, multiple research groups worldwide have focused on investigating and exploring possibilities to construct and test novel LHPs and improve their performance or develop new LHP manufacturing methods.

Despite the advantages of flat LHPs, there still exist various technical problems and challenges in their development such as:

− Sensitivity to internal pressure the internal pressure causes stress, deformation and consequently ballooning of the evaporator wall and wick and deterioration of the heating surface contact and loss of thermal connection between the heat input wall and the wick;
− Increased heat leakage from the evaporator heating zone and sidewall into the compensation chamber (CC), which results in the increase of the CC temperature and consequently the LHP resistance and frequent failures in the start-ups;
− Increased heat leakage through the wick into the liquid bore, causing the increased temperature of the liquid being supplied to the evaporator and consequently failure of start-ups;
− Increased possibility of reverse flow of vapor through the joints between the wick and casing into the compensation chamber and/or through the wick in applications where the wick thickness is reduced;
− The difficulty of sealing the casing/wick structure with long edges needs a special mechanical treatment. This causes leakage of the installation in long term maintenance and consequently the failure of the flat evaporator LHP’s operation and limits the use in space and terrestrial applications.

This review presents current endeavors to improve flat LHP performance and/or overcome the above-presented challenge. This includes:

− The creation of novel wick properties or construction techniques that improves the heat transfer capability of the overall LHP, decrease the effect of heat leak through the wick to CC, improve LHP operation reliability and stability, improve the start-up time at low operating temperatures or low operating power, overcome deformation of the evaporator and maximize heat transfer distance. Furthermore, innovative wicks can strongly enhance the LHP heat transfer performance, thermal conductivity as the wicks have greater wettability. The wick treatment improves hardness and hence prevent deformation of flat evaporator LHPs;
− Utilization of novel LHP manufacturing techniques (i.e., AM) allows the development of efficient devices with complex geometry and high surface area to volume ratio (A/V) in order to maximize the interaction between the heat source and heat sink or to maximize the surface area for evaporation/condensation processes and fabrication of products with a lower cost-to-complexity ratio and quicker production time compared to other manufacturing processes and gives the possibility of producing customized and complex freeform shapes, which are in LHPs. Furthermore, the utilization of novel LHP manufacturing techniques overcomes the above-presented challenge of sealing casing/wick that causes leakage and consequently the failure of the LHP;
− The creation of novel wick materials helps to reduce the parasitic heating from the evaporator heating zone and sidewall into CC and hence improves the LHP start up-time;
− The selection of novel working fluids (i.e., nanofluids) significantly improves the heat transfer performance of the LHP;
− The modification of the construction of a flat evaporator LHP may overcome start-up difficulties caused by the temperature overshoot at the start-up period, especially at low heat loads and might reduce or even eliminate a parasitic heal leakage in the evaporator;
− The utilization of novel manufacturing techniques increases the potential of LHP miniaturization and the possibility for dissipating high heat fluxes to take advantage of the passive cooling systems for electronic devices in multiple applications.

## Figures and Tables

**Table 1:** Comparison between recent works related to bi-porous wick LHPs.

Research Group	Working Fluid	Evaporator Casing Material	Evaporator Dimensions	Power	Maximum Heat Flux	Thermal Resistance	Wick	Heat Transport Distance	Effect
Li et al. 2012 [18]	Methanol	Copper	Ø74 mm × H28 mm	40 W–100 W	10.4 W/cm^2^	N/A	Nickel powder; Three kinds of pores: the first one is made by pore former, and mean diameter is about 120 µm; The second one is the gap between nickel powders, mean diameter is less than 2 µm; The third one is pore generated by numbers of nickel powder agglomeration, mean diameter is about 10 µm;	300 mm	Flat LHP can start-up and run successfully without dry out; The bi-porous wick can allow the LHP system to run stably and reliably; The LHP with bi-porous wick can dissipate heat load effectively and stably.
Chen et al. 2012 [17]	Ammonia	Stainless-steel	Ø43 mm × H15 mm	2.5 W–130 W	12.8 W/cm^2^	0.33 °C/W	Nickel powder; Two kinds of pores: the first one is made by a pore former, and mean diameter is about 106 µm; The second one is the gap between nickel powders, mean diameter is 4.4 µm to 5.6 µm;	335 mm	The LHP could start-up at low heat loads; The evaporator surface has very high isothermally; No obvious temperature oscillation and overshoot phenomena were observed during start-up and operation with variable heat load.
Liu et al. 2012 [14]	Methanol	Brass	Ø74 mm × H28 mm	20 W–160 W	16.8 W/cm^2^	0.46 °C/W	Primary wick: Sintered nickel powder; Secondary wick: Several hundred layers of stainless steel mesh;	300 mm	LHP can start-up at low heat load; The start-up time of LHP decrease with the increase of heat load applied to the evaporator; e.g., Q = 10 W, t_start-up@10W_ = 20 min; Q = 100 W t_start-up@100W_ = 3 min.
Wu et al. 2015 [19]	Ammonia	N/A	Ø16 mm × L65 mm	50 W–800 W	N/A	0.094 °C/W	Sinter nickel powder; Small pore diameters were around 7 µm with a range of around 1–10 µm; PMMA diameter 250 µm to 297 µm;	470 mm	Higher content of PMMA particles corresponds to better LHP performance, but beyond a maximum and optimal content the LHP performance worsens; Larger powder size leads to better vapor transport and evaporation, but beyond a certain point the large pores can cause weakened wick structure; The bi-porous wick performance was enhanced.
Kumar et al. 2018 [20]	Ethanol	Copper	25 mm × 30 mm × 10 mm	7 W–17 W	11 W/cm^2^	N/A	Naphthalene as the pore former. The average particle diameter of the copper and naphthalene powder is determined using the imaging method which is found≈10 ± 6μm and 7.5 ± 4μm, respectively;	N/A	Porosity and permeability increase with an increase in pore former content and decreases with an increase in sintering temperature; Liquid-vapor interface in the bi-porous wick moves away from the heated fin as heat load increases; The evaporative heat transfer coefficient decreases with an increase in heat load; Bi-porous wicks provide better thermal performance than mono-porous wick.
Zhang et al. 2020 [21]	Ammonia	Stainless steel	Ø60 mm × H25 mm	2.5 W–180 W	10.8 W/cm^2^	0.252 °C/W	Nickel powder; Pore diameter was about 5.8μm. The small pores were formed by the nickel particles and the large pores were formed by dissolving the pore formers.	1600 mm	LHP could start-up successfully at a low heat load; During the start-up and variable heat load tests, no unfavorable temperature overshoot or pulsation was observed; Variable conductance mode and constant conductance mode were observed at the whole range of heat load.

**Table 2:** Comparison between recent works of using AM technology in manufacturing LHP’s.

Research Group	Evaporator Casing Material	Evaporator Dimensions	Power	Thermal Resistance	Wick	Heat Transport Distance	Effect
Esarte et al. 2017 [26]	Copper	Volume 2827 mm^3^ Active length 23.2 mm	57 W, 120 W	0.15 °C/W	Stainless steel Pore radius 80 µm	100 mm	Controls the geometric size of the internal wick passages, aiming to achieve an optimal design according to the specified requirements;
Anderson et al. 2017–2021 [11,12,27,28]	Stainless steel	Ø25.4 mm × L10.16 mm	5 W– 350 W	0.13 °C/W	Stainless steel Pore radius 4.9 µm	N/A	The LHP was able to operate at low powers, against gravity, during rapid changes in heat input power and survive transients; Significant cost advantages to traditional LHP fabrication techniques; Eliminated the knife-edge seal to improve reliability;
Hu et al. 2020 [29]	Stainless steel	Flat dish Ø42 mm × H2 mm	20 W– 160 W	0.031 °C/W	Stainless steel Pore radius 100 µm	150 mm	Fast start-up; Low evaporator wall temperature during the operation;

**Table 3:** The comparison between different attempts of heat leak prevention by using non-metallic or composite wicks.

Research Group	Wick	Working Fluid	Casing Material	Evaporator Dimensions	Power	Maximum Heat Flux	Thermal Resistance	Heat Transport Distance	Effect
Wu et al. (2015) [51]	Sintered PTFE (polytetrafluoroethylene) Pore radius of 1.7 µm, the porosity of 50%, and permeability of 6.2 × 10^−12^ m^2^	Ammonia	Aluminium	L65 mm × Ø12.5 mm	600 W	$38.21\frac{W}{{\mathrm{cm}}^{2}}$	0.145 °C/W	470 mm	Low thermal conductivity to reduce the heat leakage during operation; Short wick manufacturing time; PTFE wicks are lighter than metal wicks and can be a substitute in situations with weight restrictions;
Wu et al. (2017) [46]	Sintered PTFE (polytetrafluoroethylene) Pore radius of 1.8 µm, porosity of 49%, and permeability of 5.3 × 10^−12^ m^2^	Water + Butanol aqueous solution to form self-rewetting fluid	n/a	L65 mm × Ø15.5 mm	400 W	$20\frac{W}{{\mathrm{cm}}^{2}}$	0.32 °C/W	470 mm	Adding butanol in water to form self-rewetting fluid can make water usable in LHP with sintered PTFE wicks, making the system operates successfully;
Santos et.al. (2010) [43,44]	Ceramic porous wick Pore radius 1–3 µm, porosity of 50%, and permeability of 35 × 10^−15^ m^2^	Acetone and Water	Stainless steel	L25 mm × Ø10 mm	25 W	$3.18\frac{W}{{\mathrm{cm}}^{2}}$	5.3 °C/W	260 mm and245 mm	The ceramic porous wick is a reliable alternative for LHP applications;
He et al. (2020) [49,50]	Sintered nickel wick Pore radius 3–10 µm, the porosity of 70% and permeability of 2.39 × 10^13^	R245fa	Composite copper and stainless steel	L80 mm × W80 mm × H21 mm	150 W	$13.04\frac{W}{{\mathrm{cm}}^{2}}$	n/a	270 mm	Composite-material can overcome the disadvantages of heat leak through the evaporator sidewall and easy deformation of the flat evaporator; The composite-material evaporator can effectively reduce the parasitic heating through the evaporator sidewall, forming a larger temperature difference between the evaporator back and the evaporator outlet.
Xin et al. (2018) [48]	Composite wick having different effective thermal conductivities—higher thermal conductivity on the side close to the vapour Channels and lower thermal conductivity on the side close to the liquid in the compensation chamber The outer layer (pure nickel) pore radius 5 µm (85.6%), porosity 51.3% The inner layer (Ni–10 wt% Cu) Pore radius 5 µm (68.3%), porosity 51.3%	Ammonia	n/a	L40 mm × Ø20.5 mm	10 W	$0.388\frac{W}{{\mathrm{cm}}^{2}}$	n/a	260 mm	Composite wick as a whole has a lower heat leak compared to the sintered pure nickel wick and presents better performance. The different arrangement of thermal conductivity in the wick improved heat transfer performance in the LHP with the composite wick

**Table 4:** Comparison between recent works of using nanofluids in LHP.

Research Group	Nanofluid	Evaporator Casing Material	Evaporator Dimensions	Power	Maximum Heat Flux	Thermal Resistance	Wick	Heat Transport Distance	Effect
Gunnasegaran et al. 2013 [53]	Silica nanofluid (SiO_2_–H_2_O)	Copper	L50 mm × W50 mm × H4 mm	20 W–100 W	$4\frac{W}{{\mathrm{cm}}^{2}}$	1.304 °C/W	Mesh Size—n/a	830 mm	The total thermal resistance of LHP decreases when using SiO_2_–H_2_O nanofluid compared with pure water;
Putra et al. 2014 [54]	Al_2_O_3_-Water	Copper	Tube Ø 20 mm and 100 mm in length	10 W–30 W	$0.47\frac{W}{{\mathrm{cm}}^{2}}$	0.68 °C/W	Porous biomaterial (Collaria) mean pore diameters a 83 µm, 56 µm, 170 µm, 124 µm	270 mm	The total heat resistance of the LHP was lowest when using a biomaterial wick; The application of the biomaterial wick can decrease the thermal resistance;
Wan et al. 2015 [55]	Cu-Water	Copper	L55 × W50 × H18	25 W–150 W	$5.45\frac{W}{{\mathrm{cm}}^{2}}$	0.065 °C/W	Porous copper wick mean pore diameter 65 µm	350 mm	Start-up time and the transient stage are reduced; The evaporator wall temperature and the total thermal resistance de crease; The evaporation heat transfer coefficient of the LHP increases when using the nanofluid;
Tharayil et al. 2016 [56]	Graphene–water	Copper	L20 mm × W20 mm × H7.5 mm	20– 380 W	$95\frac{W}{{\mathrm{cm}}^{2}}$	0.083 °C/W	Screen mesh wick (100 mesh) 0.25 mm 7 layers	127 mm	Improved the heat transfer performance; The thermal resistance of LHP decreases with anincrease in nanofluid;

## Data Availability

The data supporting reported results can be found at the website ScienceDirect.com (scientific internet browser) udder the references listed below.
