# Correction: Szymanski et al. Recent Advances in Loop Heat Pipes with Flat Evaporator. *Entropy* 2021, *23*, 1374

**DOI:** 10.3390/e24020162

**Published:** 2022-01-21

**Authors:** Pawel Szymanski, Richard Law, Ryan J. MᶜGlen, David A. Reay

**Affiliations:** 1 Faculty of Mechanical Engineering and Ship Technology, Gdansk University of Technology, 80-233 Gdańsk, Poland; 2 School of Engineering, Newcastle University, Newcastle Upon Tyne NE1 7RU, UK; Richard.law2@newcastle.ac.uk (R.L.); David.reay@newcastle.ac.uk (D.A.R.); 3 Aavid, Thermal Division of Boyd Corporation, Ashington NE63 8QW, UK; Ryan.McGlen@boydcorp.com

The author of the original publication [1] wishes to make the following corrections to the Authorship, Author Contributions, and Funding Information: 

Richard Law, Ryan J. MᶜGlen, and David A. Reay have been added to the list of contributing authors. The updated Author Contributions statement is provided below:

**Author Contributions:** P.S.: conceptualization, formal analysis, investigation, methodology, writing—original draft, writing—review and editing; R.L.: conceptualization, formal analysis, project administration, resources, investigation, methodology, supervision, writing—review and editing, funding acquisition; R.J.M.: conceptualization, formal analysis, resources, investigation, methodology, supervision, writing—review and editing, funding acquisition; D.A.R.: conceptualization, formal analysis, resources, investigation, methodology, supervision, writing—review and editing. All authors have read and agreed to the published version of the manuscript.

The authors wish to update the missing Funding statement as follows:

**Funding:** This work was funded by the UK EPSRC, Newcastle University, the Aavid Thermal Division of Boyd Corp under a CASE PhD studentship, and the Dean of the Faculty of Mechanical Engineering and Shipbuilding, Gdansk University of Technology, Poland.

The authors apologize for any inconvenience caused and state that the scientific conclusions are unaffected. The original publication has been updated.
